# Conductive framework of inverse opal structure for sulfur cathode in lithium-sulfur batteries

**DOI:** 10.1038/srep32800

**Published:** 2016-09-07

**Authors:** Lu Jin, Xiaopeng Huang, Guobo Zeng, Hua Wu, Massimo Morbidelli

**Affiliations:** 1 Institute for Chemical and Bioengineering, Department of Chemistry and Applied Biosciences, ETH Zurich, 8093 Zurich, Switzerland; 2 Laboratory of Microsystems, Institute of Microengineering, School of Engineering, EPFL, 1015 Lausanne, Switzerland; 3 Laboratory for Multifunctional Materials, Department of Materials, ETH Zurich, 8093 Zurich, Switzerland

## Abstract

As a promising cathode inheritor for lithium-ion batteries, the sulfur cathode exhibits very high theoretical volumetric capacity and energy density. In its practical applications, one has to solve the insulating properties of sulfur and the shuttle effect that deteriorates cycling stability. The state-of-the-art approaches are to confine sulfur in a conductive matrix. In this work, we utilize monodisperse polystyrene nanoparticles as sacrificial templates to build polypyrrole (PPy) framework of an inverse opal structure to accommodate (encapsulate) sulfur through a combined *in situ* polymerization and melting infiltration approach. In the design, the interconnected conductive PPy provides open channels for sulfur infiltration, improves electrical and ionic conductivity of the embedded sulfur, and reduces polysulfide dissolution in the electrolyte through physical and chemical adsorption. The flexibility of PPy and partial filling of the inverse opal structure endure possible expansion and deformation during long-term cycling. It is found that the long cycling stability of the cells using the prepared material as the cathode can be substantially improved. The result demonstrates the possibility of constructing a pure conductive polymer framework to accommodate insulate sulfur in ion battery applications.

Developing high-performance rechargeable batteries is critical for utilizing the renewable energies to apply in portable electronic devices, green electric vehicles and large scale grid energy storage system. The ever-increasing research interests in lithium-ion batteries (LIBs) have been widely boosted since last decades owing to their most viable near-term commercialization^1^,^2^. In spite of the popularity of LIBs, the practical electrochemical properties (e.g., energy density and volumetric capacity) of LIBs are anticipated to approach the ceiling due to the inherent limitations derived from Li^+^ insertion/extraction chemistry. At the same time, cost and safety are also necessarily considered for large-scale applications. New directions are inspired on creating next generation Li-ion systems, among which lithium-sulfur (Li-S) batteries provide a promising alternative owing to its advantages over conventional LIBs in terms of high theoretical specific capacity of 1673 mAhg^−1^ and energy density of 2600 Whkg^−1^, which are five folds as those of state-of-the-art LIBs^3^,^4^. Additionally, the elemental sulfur is an abundant and inexpensive resource in nature, benefiting considerable cost cutting in widespread applications^5^.

However, the Li-S batteries intrinsically suffer from several challenges that impede their practical utilization^6^,^7^. The electronic and ionic insulating nature of sulfur and the discharged product Li_2_S restrict the utilization of active materials; the dissolution of intermediate species, polysulfide, in commonly used electrolytes leads to parasitic reaction with lithium anode and shuttle effect in charge-discharge process; volumetric expansion of sulfur results in pulverization of cathode materials and further deteriorates the cycling performance of the cell.

Various strategies have been explored to address these shortcomings, which can be wrapped up to two categories. The first strategy is to incorporate sulfur into a porous conductive carbon framework. As a common conductive support, carbon can constrain the sulfur growth inside its exquisite structure and provide adequate electrical contact to improve the electrical and ionic conductivity. Nazar *et al*. reported a conductive mesoporous carbon framework (CMK-3) to uniformly accommodate nearly 70 wt% of sulfur nanofiller, through which a maximum reversible capacities of 1320 mAh g^−1^ was achieved^8^. Liang *et al*. proposed a low-cost, truly green and recyclable sheet-like carbon material, shaddock peel carbon sheets (SPCS) with large surface area and high conductivity, with which after the sulfur infiltration the battery reaches a high reversible capacity of 722.5 mAh g^−1^ at 0.2 C after 100 cycles^9^. A remarkable design of framework attracting much attention is inverse opal structure, which offers high ordered arrangement, high loading ability (74% of the theoretical volume content), simple synthesis route and high controllability (from tens of nanometers to several micrometers). Recently, McNulty *et al*. developed inverse-opal full battery with paired intercalation-typed cathode (V_2_O_5_) and conversion-typed anode (Co_3_O_4_), which exhibited superior cycling performance^10^. Doherty *et al*. used PMMA colloidal crystal templates to produce hierarchical porous, open lattice cathode of LiFePO_4_^11^. Huang *et al*. entrapped electrode active nanoparticles in an interpenetrating macroporous carbon inverse opal and demonstrated enhanced ion and electron transport kinetics^12^. Agrawal *et al*. developed a route to synthesize porous carbon for loading sulfur based on resorcinol formaldehyde replicated from PMMA opal structure^13^. However, nonpolar carbon framework has been proved inefficient in attaching polar Li_x_S on its surface during discharge process, leading to capacity fading^14^,^15^.

The other approach is to build core-shell (or yolk-shell) structure with sulfur as the core (or yolk) and other active component as the shell, including inorganic (e.g., carbon, TiO_2_) and polymer materials^14^,^16^,^17^,^18^,^19^. Cui *et al*. designed a sulphur-TiO_2_ yolk-shell nanoarchitecture to accommodate the volume expansion of sulfur and to minimize polysulfide dissolution, showing a capacity decay of 0.033% after 1000 cycles^20^. Polyvinylpyrrolidone^21^ and polydopamine^22^ are also reported to be interesting shell materials for sulfur with improved electrochemical performance. Particularly, conducting polymers are proved to be exceptional candidates which can improve the confinement of polysufides and meanwhile maintain an excellent electrochemical performance due to its conducting and flexible nature. Nevertheless, compared to the framework-based strategy, the sulfur encapsulation capability of shell-based approach is difficult to be guaranteed.

Herein, we design a conductive polypyrrole (PPy) framework to accommodate and encapsulate sulfur. The selection of PPy to support sulfur is mainly based on the following considerations. Firstly, compared with pure carbon-based framework that has poor adsorption ability for polysulfide species, the nitrogen-rich conductive backbone (PPy) is demonstrated to induce chemical adsorption of sulfur; thus it could provide more active sites and facilitate the charge and ion transport^23^. Secondly, the accessibility of electrolyte is also improved within nitrogen-rich PPy matrix^24^. Thirdly, the PPy framework is mechanically more flexible (elastic) compared with the carbon framework. Thus, the structural deformation resulting from sulfur expansion could be alleviated, and consequently cycling performance is stabilized^14^,^25^,^26^. Moreover, PPy is electrochemically active for lithium intercalation/extraction^27^. To realize well-ordered inverse opal PPy framework, we use highly monodispersed polystyrene (PS) nanoparticle (NP) array as template, followed by the removal of PS template and infiltration of the sulfur element. The electrochemical performance is characterized to demonstrate the successful physical confinement and chemical adsorption of polysulfide in this work.

## Results and Discussion

### PPy Framework of Inverse Opal Structure

**Figure 1:**
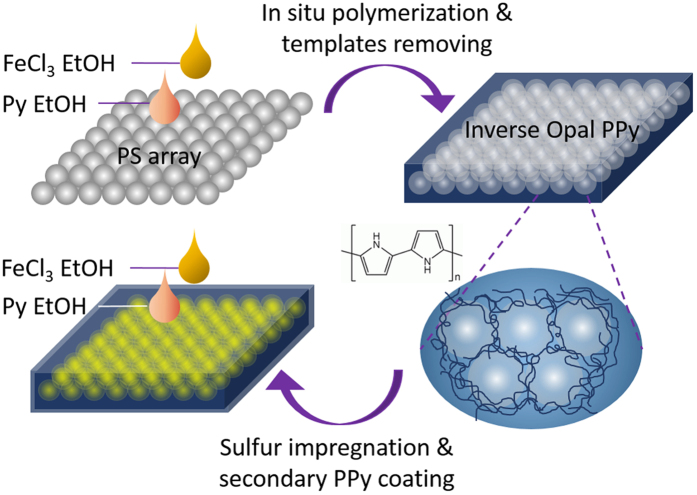
Synthesis procedure of PPy/S/PPy cathode material. Monodisperse PS nanoparticles are prepared and self-assemble to form ordered array. Sequential infiltrations of ethanol solutions of FeCl_3_ and pyrrole are applied and *in situ* polymerization occurs to form PPy framework. After removal of PS templates, melted sulfur infiltrates, and a secondary PPy coating is applied.

As illustrated in Fig. 1, the inverse opal structural PPy frame-work is prepared based on the sacrificial template. Monodispersed PS NPs with tunable size are simply prepared through dispersion polymerization, and then self-assemble to form the ordered array. After complete evaporation of liquid from the surface, the ethanol solutions of oxidant, FeCl_3_, and pyrrole are sequentially added to soak the PS NP array, and *in situ* polymerized. Naturally, the formed conjugated PPy fills the vacancies inside the PS NP array. Afterwards, the PS template is completely removed by immersing the entire composite in dichloromethane, while the PPy network retains and forms the inverse opal structure, leaving the space for subsequent infiltration of melted sulfur.

**Figure 2:**
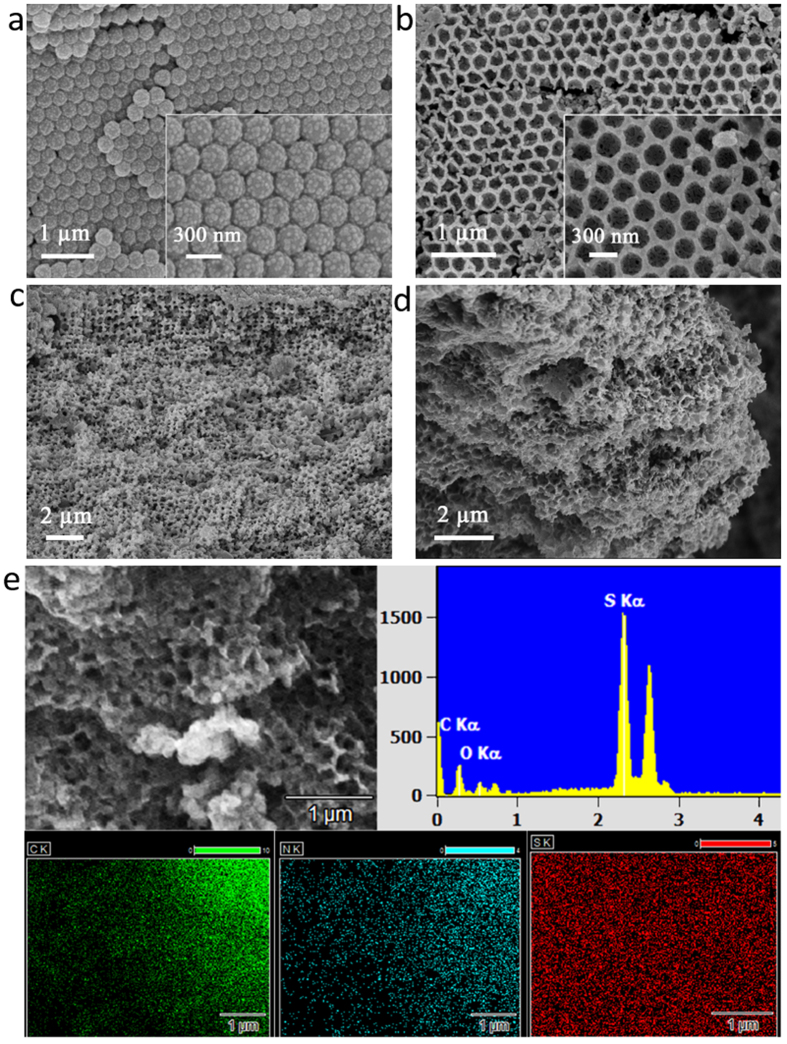
SEM images of (**a**) PPy based on PS template; (**b**) PPy framework of inverse-opal structure; (**c**) PPy framework after sulfur impregnation; (**d**) PPy/S/PPy cathode after secondary PPy coating. (**e**) Elemental mapping of the PPy/S/PPy cathode (C, N and S distributions are qualitatively represented in green, blue and red, respectively).

The morphologies of the PS NP array and the inverse opal PPy framework are characterized by scanning electron microscopy (SEM), as shown in Fig. 2. By varying the SDS amount, monodispersed PS NPs with average diameter of 170 and 300 nm, respectively, are acquired. After *in situ* polymerization, PPy covers the surface of the PS NPs and fills the voids among them, as verified by Fig. 2a. After removing the PS templates by solvent dissolving, the PPy framework remains the hexagonal porous structure, as shown in Fig. 2b. The pores where the PS NPs are originally located are interconnected *via* the PPy backbone, forming an integral conductive scaffold. These pores provide sufficient space for accommodating sulfur, enduring its volume expansion during discharging, and improving the overall conductivity. In addition, it is expected that at the contacting points between the PS NPs in the array, the PPy thickness reduces to the minimum, even leading to formation of open nanoscale channels. Such open channels facilitate impregnation of sulfur in the next step, as well as permeation of electrolyte, thus Li-ion transport. Furthermore, the PPy scaffold can physically and chemically adsorb the polysulfide, thus reducing its dissolution in the electrolyte and consequently improving the cycling performance^14^,^28^,^29^.

### Sulfur Impregnation

To impregnate sulfur into the inverse opal PPy framework (see Fig. 1), a melting diffusion strategy is adopted. In particular, the impregnation was performed at 155 °C, at which the melted sulfur possesses the minimum viscosity^30^. The impregnation process is facilitated and promoted by several factors such as porosity and possible open channels of the PPy framework, the hydrophobic interactions between PPy and sulfur, etc. Fig. 2c shows a SEM picture of the obtained sulfur-in-PPy material, referred to as PPy/S, where the PPy framework is still evident, but the holes are now partially filled with sulfur. Since there are inevitably some free-standing sulfur granules on the surface, to encapsulate them, a second layer of PPy is conducted through again *in situ* polymerization, leading to the final cathode material, referred to as PPy/S/PPy. Note that the mass ratio between PPy and S before heat infiltration is designed to be 1:2. As shown in Fig. 2d, after the secondary PPy coating, the ordered structure of the PPy framework becomes less observable. The results of elemental mapping using EDX for the obtained PPy/S/PPy are shown in Fig. 2e. It can be seen that the signals of carbon, nitrogen and sulfur are evenly exhibited on the whole inverse opal framework, indicating the homogeneous distribution of sulfur in the PPy framework.

**Figure 3:**
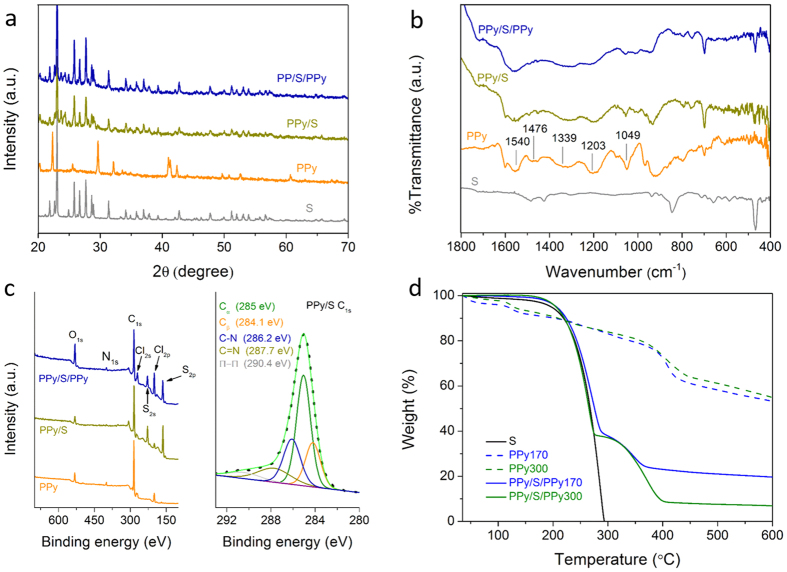
Characterization of the PPy/S/PPy300 sample with (**a**) XRD, (**b**) FTIR and (**c**) XPS, and (**d**) TGA of pure sulfur, PPy framework and PPy/S/PPy samples.

Figure 3a shows the X-ray diffraction (XRD) patterns of pristine sulfur (black curve), PPy synthesized using 300 nm PS NPs as template (red curve), PPy/S and PPy/S/PPy. The spectrum of pristine sulfur matches the reported orthorhombic crystal type^31^. The presence of sulfur in PPy/S and PPy/S/PPy is clearly evidenced by comparing the spectra with that of pristine sulfur. Note that in spite of existence of PPy, PPy/S and PPy/S/PPy show the same peaks as those of sulfur. In addition, the spectra of PPy/S and PPy/S/PPy are very similar, indicating that no significant change in the spectrum occurs after the secondary PPy coating. These XRD results indicate that the encapsulated sulfur within the PPy framework remains its high crystallinity.

To confirm the presence of PPy in PPy/S and PPy/S/PPy, Fourier transform infrared (FTIR) spectroscopy has been used, and the recorded spectra are shown in Fig. 3b. The spectrum of PPy (red curve) shows typical bands at 1540 and 1476 cm^−1^ standing for pyrrole ring, bands at 1339 and 1049 cm^−1^ owing to the =C–H in-plane vibrations, and band at 1203 cm^−1^ owing to the C–N stretching vibration. For PPy/S and PPy/S/PPy, the bands arise at positions similar to those appearing in pure PPy, and meanwhile some spectral features of sulfur are also preserved. X-ray photoelectron spectroscopy (XPS) has been also applied to qualitatively measure the changes in surface chemistry. As shown in Fig. 3c, typical peaks of C1s (285.0 eV) and N1s (400.3 eV) are attributed to carbon and nitrogen of PPy. Peaks of Cl_2s_ (267.0 eV) and Cl_2p_ (199.0 eV) arise from the residues of FeCl_3_. For PPy/S, S_2s_ (228.0 eV) and S_2p_ (164.0 eV) peaks of elemental sulfur are detectable, while the N and Cl signals are slightly weakened. For PPy/S/PPy with the secondary PPy coating, the peaks of N_1s_ and Cl_2s/sp_ become stronger compared with those of S_2s_ and S_2p_, suggesting more PPy component on the surface. The changes in XPS confirms that the obtained materials are in conformity with the design. Additionally, the C_1s_ main peak in PPy/S is deconvoluted to five peaks, corresponding to α-C and β-C signals, C-N and C = N covalent bonding on pyrrole ring, and π-π conjugated structure on PPy backbone, respectively. It should be mentioned that the peak at 268.2 eV mainly attributes to the signal from imine bonding (C = N), but also possibly from C–S bonding^32^.

Two PPy/S/PPy samples have been prepared using the PS NPs of 170 and 300 nm as template, respectively, referred to as PPy/S/PPy170 and PPy/S/PPy300. The total sulfur content in the samples is determined by thermogravimetric analysis (TGA) in N_2_ flow from 35 to 600 °C, as shown in Fig. 3d. As references, TGA is also performed for pristine sulfur and PPy. For sulfur, the weight decays sharply and is completely exhausted before 300 °C, while for PPy the weight decays much slower, but a slight steep weight loss appears around 400 °C, which could be attributed to residuals of the PS template. Both the PPy/S/PPy samples show a two-step weight loss profile. The first step lying in temperature smaller than 300 °C corresponds to sulfur consumption, and the second step is attributed to the gradual depletion of PPy. Therefore, using TGA of pristine sulfur as the background, we can easily estimate the sulfur content in PPy/S/PPy170 and PPy/S/PPy300, which in both cases is approximate 61 wt%. From our initial design, the sulfur content should be 66 wt%. The small difference is mostly due to mass contribution from secondary PPy coating or a certain part of sulfur evaporation. Now, let us approximately estimate the volume fraction occupied by the infiltrated sulfur in the inverse opal structure. Since our PS particles are rather monodisperse and their array is rather ordered, we can assume that the inverse opal structure is generated by close-packing of equal spheres. In this case, the initial void fraction can be estimated to be 74% (see Supplementary Information, SI), and it follows that the volume occupied by PPy is 26%. Then, based on the sulfur weight fraction, 61%, and the density of sulfur and PPy (2.07 and 1.48 g cm^−3^)^33^,^34^, the volume occupied by sulfur is 29.1%, or in other words, the infiltrated sulfur has occupied only 39.3% of the initial void in the inverse opal structure. After discharging, assuming that the sulfur is completely converted to Li_2_S, with the Li_2_S density (1.66 g cm^−3^), we can estimate that 70.4% of the initial void have been occupied by total Li_2_S. Thus, even though this evaluation corresponds to the ideal case, it confirms that the space is enough for accommodating volume expansion. The above results indicate that using the present approach, one can quantitatively control the filling level of sulfur inside the template by pre-defining the mass ratio between sulfur and PPy.

### Electrochemical Properties and Cycling Performance

**Figure 4:**
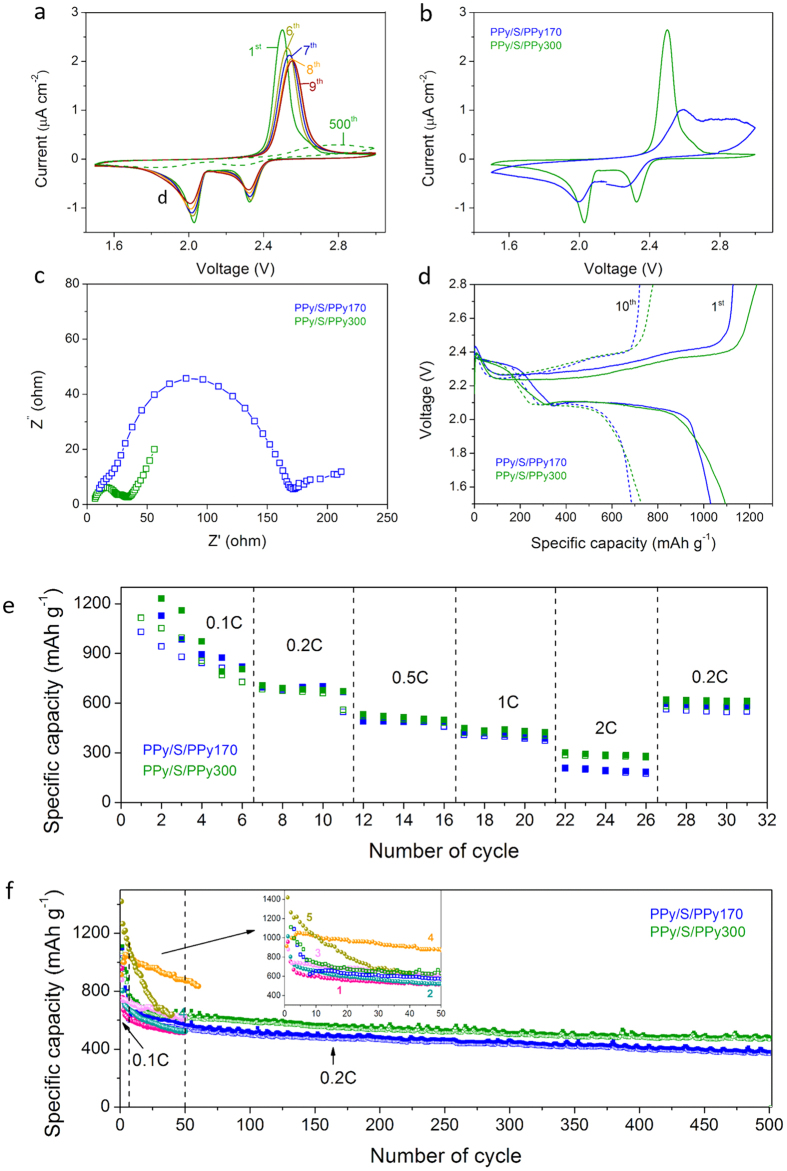
Electrochemical characterization: (**a**) voltammograms (CV) of selected initial five cycles of PPy/S/PPy300 cathode, and (**b**) the 2^nd^ cycle of PPy/S/PPy170 and PPy/S/PPy300 at a sweep rate of 0.1 mVs^−1^, (**c**) impedance analysis, (**d**) 1st and 10th discharge and charge voltage profiles in 1.5–2.8 V at 0.1C rate, (**e**) rate performance and (**f**) cycling performance of the two cathodes. Curves 1 to 5 are cycling performances reproduced from Fu & Manthiram^25^, Fu & Manthiram^38^, Fu *et al*.^39^, Wang *et al*.^18^ and Zhang *et al*.^40^, respectively.

The electrochemical properties and cycling performance of the developed PPy/S/PPy cathode materials are investigated by testing the assembled coin cells. Details about the assembly is given in Section, Methods. Figure 4a shows the voltammograms (CV) of the initial few cycles for the cell made of PPy/S/PPy300. The CV curves exhibit the typical shape of two separated cathodic peaks and a single anodic peak. The cathodic peaks at 2.35 and 2.05 V are assigned to the conversion of high order lithium polysulfide (Li_2_S_8_) to low order species (Li_2_S_x_, 4 <×< 8) and further to solid-state Li_2_S_2_/Li_2_S. The anodic peak at 2.5 V can be ascribed to the kinetically favorable conversion of lithium sulfide to S_x_^2−^ (2 ≤ × ≤ 8) species^4^. The detailed redox reactions occurring at cathode are shown in SI.

The peaks remain constant upon the scanning rate of 0.1 mV/s, suggesting excellent electrochemical stability of the material due to the efficient encapsulation of sulfur in the PPy framework. In Fig. 4b, the CV curve is compared with that of PPy/S/PPy170, and for the latter the peaks are much broader. This may indicate that the sulfur encapsulation efficiency is lower for PPy/S/PPy170, and significant amount of sulfur uncovered by PPy results in shuttle effect due to the dissolvable polysulfide species. This is consistent with the electrochemical impedance spectra shown in Fig. 4c, where PPy/S/PPy170 exhibits a much larger semicircle, indicating much higher charge transfer resistance^35^. This may indicate that the open channels generated at the contact among the PS (template) NPs are essential for an efficient sulfur encapsulation. It is expected that the size of such open channels decreases as the size of the PS NPs decreases. Thus, in the case of PPy/S/PPy170, the smaller dimension of the open channels leads to the lower encapsulation efficiency.

Charge/discharge behaviors of the two cells are also investigated, and the voltage profiles are shown in Fig. 4d. In general, the two plateaus of discharge curve and one extended plateau of charge curve match well with the redox peaks of the CV curves. At 0.1C rate (1C = 1673 mA g^−1^), the initial discharge specific capacities of PPy/S/PPy300 and PPy/S/PPy170 achieve 1094 and 1030 mAh g^−1^, respectively. During the following cycles, significant capacity decay is observed. After 10 cycles, the specific capacities decrease to 727 and 685 mAh g^−1^. This indicates that for both PPy/S/PPy300 and PPy/S/PPy170, part of the sulfur was not encapsulated, which was irreversibly dissolved in electrolyte. Then, the rate performance for the two cells was tested under various current densities from 0.1C to 2C, as shown in Fig. 4e. In general, as the applied current increases, the specific capacity of the two cells decreases, but it resumes when the current starts to increase. The difference between the two cells is insignificant in the range of the current density from 0.1C to 1C. Further increasing the current density, we have observed considerable difference in the specific capacity between PPy/S/PPy300 and PPy/S/PPy170, as in the case of 2C in Fig. 4e. This is consistent with impedance analysis in Fig. 4c, which suggests larger charge transfer resistance in the case of PPy/S/PPy170.

However, for prolonged cycling, as shown in Fig. 4f, the difference between the two cells is significant. In particular, both cells are plagued with initial capacity loss of approximately 20% after the first cycle at the current density of 0.1C. This is again mainly related to the small part of the sulfur that was not encapsulated, which, when formed Li polysulfide, was irreversibly dissolved in electrolyte^36^,^37^. With ongoing cycling at 0.2C, the capacity fading is still significant during the initial 50 cycles, but remains more constant till 500 cycles. The good performance in the long cycling stability obviously benefits from the sulfur encapsulation by the PPy/S/PPy structure, where the elemental sulfur and polysulfides are strongly confined physically and chemically^29^. Detachment of Li_2_S from the conductive matrix is the main factor causing the capacity fading. Thus, efficient sulfur confinement in a stretchable and robust host matrix is crucial to improve the cycle stability. In addition, since the volume expansion during discharging occurs substantially for Li-S batteries, deformation and collapse of the supporting framework are typically the main issues. The results in Fig. 4f confirm that the inverse opal PPy scaffold is stable and flexible, favorable for accommodating stress and volume variation of sulfur and thus maintaining good cycling stability, which is consistent with the results in the literature for various conformations of conductive polymers^14^,^26^,^28^. For the long cycle tests in Fig. 4f, the specific capacity curve of the cell with PPy/S/PPy300 is always above that with PPy/S/PPy170, and after 500 cycles, it is 485 mAh g^−1^ for the former and 380 mAh g^−1^ for the latter. This further confirms that the sulfur encapsulation efficiency is higher for PPy/S/PPy300 than for PPy/S/PPy170.

We have also compared our results with those reported in literature under similar conditions, as shown in Fig. 4f with curves 1 to 6. Fu *et al*. have constructed polypyrrole-sulfur cathode materials with different core-shell structures and their performances within 50 cycles are re-produced in Fig. 4f as curves 1 to 3^25^,^38^,^39^. In some cases, additional components such as MWCNT (Wang *et al*.^18^) and graphene (Zhang *et al*.^40^) were introduced to improve the integral conductivity of PPy/S, and, as given by Curves 4 and 5, the cathode performance was indeed increased. All these results indicate that there is still large space for further enhancement of the capacity and stability of the sulfur-based cathode materials through various possible strategies.

In summary, we have used monodisperse PS NPs as sacrificial templates to have built an entire conductive, inverse opal structure with PPy to encapsulate sulfur and wrap the composite with an additional coating. The interconnected conductive PPy provides open channels for sulfur infiltration, improves electrical and ionic conductivity of the embedded sulfur, and reduces polysulfide dissolution in the electrolyte through physical and chemical adsorption. Due to the flexibility of PPy and partial filling of the inverse opal structure for compensating possible expansion and deformation, it is found that the long cycling stability of the cells using the prepared material as the cathode have been substantially improved. It should be mentioned that in some Li-ion battery applications, the inverse opal void was used as the channel for electrolyte. In this case, due to the 3D interconnected channels, high surface area and short path of the Li-ion diffusion, although the battery rate performance is very good, the tap density of the realized battery is very low, thus low interest in practical applications. This is not the case in our design, and we have used the 3D inverse opal void to accommodate the active cathode material, sulfur. As estimated in previous sections, the total volume of Li_2_S after discharging can occupy at least 70% volume of the void. In this sense, our structure is direct instead of inverse opal. In addition, all the steps involved in our preparation technique are rather conventional, and most of them are widely used at industrial scales. Thus, the proposed design of the sulfur cathode material is scalable to industrial productions.

## Methods

### Synthesis of PS Templates

Deionized water and ethanol are mixed at a volume ratio of 2:5, with sodium dodecyl sulfate (Fluka) dissolved (3, 4 wt% with respect to monomer). After injecting 8 ml of styrene monomer (Sigma Aldrich) and adding potassium persulfate (Sigma Aldrich) as initiator, polymerization starts under 75 °C and is completed after 6 h. Ion exchange resin is added into the obtained latex to remove the surfactants. The obtained latex is transferred to a petri dish, leaving the PS nanoparticles to self-assemble to an ordered array during drying process.

### *In Situ* Polymerization of PPy

After further solidifying the deposit in an oven at 80 °C for 3 h, 5 ml of 0.5 M iron (Ш) chloride (abcr, Germany) ethanol solution is dropwise added to 2 g dried PS colloidal deposit. The solution wets the entire template until it is slightly moist, followed by storing the template in 4 °C fridge for 1 h. 5 ml pre-cooled 0.5 M pyrrole (abcr) ethanol solution is dropwise added to trigger *in situ* polymerization, which is proceeding gently under 4 °C overnight. Afterwards, the PS templates are removed by immersing the composite in dichloromethane for 3 h.

### Sulfur Impregnation and Secondary Coating of PPy

The obtained PPy is dried and grinded with sulfur powder (Sigma Aldrich, 1:2, w/w) in an agate mortar for half an hour. Then the mixed powder is placed in Teflon tape sealed vial. The vial is transferred to a tube furnace to heat at 5 °C/min till 155 °C and maintained for 8 hours. The product is collected and undergoes the same procedure to *in situ* form the secondary PPy layer on the surface of the composite to ensure an efficient wrapping.

### Cathode Materials Preparation and Batteries Assembling

The powder sample is grounded sufficiently and mixed with carbon black and polyvinylidene fluoride (Sigma Aldrich, 8:1:1 in mass ratio) in N-methyl-2-pyrrolidone (abcr). The obtained slurry is coated onto the aluminum foil and dried in vacuum oven at 60 °C overnight to form homogeneous film. The pasted foil is punched to round pieces, serving as cathodes. The electrodes are tested in CR2032 coin cell with 0.5 M Lithium Bis(trifluoromethane)sulfonamide (Alfa Aersa) and 0.5 M LiNO_3_ (Alfa Aersa) in 1:1 (v/v) 1,3-dioxolane/1,2-dimethoxyethane (Sigma Aldrich), a Teflon Celgard separator, and lithium disk (Alfa-Aesar). The electrochemical performance is evaluated in Biologic instrument (VMP3) and LAND CT2001A testing system.

## Additional Information

**How to cite this article**: Jin, L. *et al*. Conductive framework of inverse opal structure for sulfur cathode in lithium-sulfur batteries. *Sci. Rep.* **6**, 32800; doi: 10.1038/srep32800 (2016).

## Supplementary Material

Supplementary Information
