# Supplementary material for: Conductive framework of inverse opal structure for sulfur cathode in lithium-sulfur batteries
Source: Sci Rep. 2016 Sep 7;6:32800. doi: 10.1038/srep32800 (PMC5013407; doi:10.1038/srep32800)
Supplement: Supplementary Information [file srep32800-s1.pdf]

Supplementary Information

Conductive framework of inverse opal structure for sulfur cathode in lithium-sulfur batteries

Lu Jin[1], Xiaopeng Huang[2], Guobo Zeng[3], Hua Wu[1] & Massimo Morbidelli[1]

[1]Institute for Chemical and Bioengineering, Department of Chemistry and Applied Biosciences, ETH Zurich, 8093 Zurich, Switzerland. [2]Laboratory of Microsystems, Institute of Microengineering, School of Engineering, EPFL, 1015 Lausanne, Switzerland. [3]Laboratory for Multifunctional Materials, Department of Materials, ETH Zurich, 8093 Zurich, Switzerland. Correspondence and requests for materials should be addressed to H.W. (email: hua.wu@chem.ethz.ch) or M.M. (email: massimo.morbidelli@chem.ethz.ch)

1. Volume fraction of close-packing of equal spheres

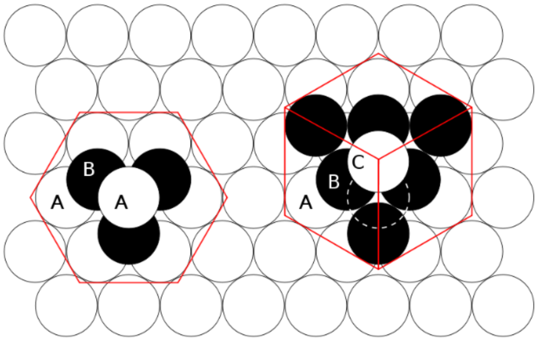

In both the alternated sequential modes of hexagonal close-packing (ABAB) and cubic close-packing (ABCABC), each sphere is surrounded by other 12 neighboring spheres, and the average density is:

$$\frac{\pi}{3\sqrt{2}} \approx 0.74$$

This basically represents the void fraction of our inverse opal structure, and it follows that the volume fraction occupied by PPy is 1-0.74 = 0.26.

2. The redox reactions occurring at cathode

The redox reactions occurring have been well documented in the literature:[1]

Reduction reaction (discharging process):

$$2Li^{+} + S_8 + 2e^{-} \rightarrow Li_2S_8 \qquad (1)$$

$$Li_2S_8 + Li^{+} + e^{-} \rightarrow Li_2S_x \ (4 \leq x < 8)$$

$$Li_2S_2 + 2Li^{+} + 2e^{-} \rightarrow 2Li_2S \qquad (2)$$

Oxidation reactions (charging process):

$$Li_2S \rightarrow Li_2S_x + Li^{+} + e^{-} \ (4 \leq x \leq 8)$$

$$2Li_2S_4 \rightarrow Li_2S_8 + 2Li^{+} + 2e^{-}$$

$$Li_2S_8 + 2Li_2S \rightarrow 2Li_2S_2 + Li_2S_6$$

$$4Li_2S_6 \rightarrow 3Li_2S_8 + 2Li^{+} + 2e^{-}$$

In Fig. 4a, the cathodic peaks appearing at 2.35 V and 2.05 V correspond to reactions (1) and (2), while anodic peak appearing at 2.5 V is related to all oxidation reactions shown above.

1 Wild, M. *et al.* Lithium sulfur batteries, a mechanistic review. *Energ Environ Sci* **8**, 3477-3494, (2015).
